# Mobile Versus Fixed-Bearing in Medial Unicompartmental Knee Arthroplasty: An Average 10-Year Follow-Up

**DOI:** 10.3390/jcm14207144

**Published:** 2025-10-10

**Authors:** Sumin Lim, Tae Hun Kim, Do Young Park, Jung Sunwoo, Jun Young Chung

**Affiliations:** Department of Orthopedic Surgery, School of Medicine, Ajou University, 164 Worldcup-ro, Yongtong-gu, Suwon 16499, Republic of Korea; khoo1003@gmail.com (S.L.); realthkim@hanmail.net (T.H.K.); doytheboy@hanmail.net (D.Y.P.); swj21@ajou.ac.kr (J.S.)

**Keywords:** osteoarthritis, arthroplasty, unicompartmental knee arthroplasty, fixed-bearing, mobile-bearing

## Abstract

**Background:** Unicompartmental knee arthroplasty (UKA) represents a well-recognized treatment option for isolated medial compartment osteoarthritis; however, the debate regarding the superiority of fixed-bearing versus mobile-bearing designs continues. We aimed to evaluate the mid- to long-term outcomes of medial UKA comparing mobile- versus fixed-bearing designs within a single institution over an average 10-year follow-up. **Methods:** This retrospective study included 81 consecutive patients who underwent primary medial UKA (45 fixed-bearing and 36 mobile-bearing) with a minimum five-year follow-up. Clinical outcomes were measured using the Western Ontario and McMaster Universities Osteoarthritis Index (WOMAC) score and range of motion (ROM). Radiological measurements included hip-knee-ankle axis angle (HKA) and osteoarthritis progression. Implant survivorship was evaluated using Kaplan–Meier analysis, with failure defined as either conversion to total knee arthroplasty (TKA) or polyethylene (PE) exchange. **Results:** At a mean follow-up of 10.6 years, WOMAC scores, ROM, and radiological outcomes showed no statistically significant differences between the fixed-bearing and mobile-bearing groups. Significantly higher failure rates were observed in the mobile-bearing group, both when considering conversion only (*p* = 0.041) and when including conversion or PE exchange (*p* = 0.009). Survival analysis demonstrated 10-year rates of 97.8% for fixed-bearing and 88.9% for mobile-bearing with TKA conversion defined as failure (*p* = 0.066). Using combined failure criteria of TKA conversion or PE exchange, 10-year survival rates were 97.8% for fixed-bearing and 83.3% for mobile-bearing (*p* = 0.015). **Conclusions:** At a mean 10.6-year follow-up, clinical and radiological outcomes were comparable, but fixed-bearing UKA demonstrated superior survivorship.

## 1. Introduction

Unicompartmental knee arthroplasty (UKA) represents an effective surgical intervention for isolated medial compartment osteoarthritis (OA), with reported survival rates reaching 95% at 10 years and 90% at 20 years [1,2]. UKA accounts for approximately 20% of all knee arthroplasty procedures [3], and the annual incidence of arthroplasty procedures continues to rise globally [4]. Furthermore, UKA offers several advantages over total knee arthroplasty (TKA), including faster recovery, reduced complications, lower mortality rates, and superior clinical outcomes, though it is associated with a slightly higher reoperation rate [5].

In medial UKA, two types of bearings are utilized: fixed-bearing and mobile-bearing designs. In fixed-bearing implants, the polyethylene (PE) insert is securely fixed to the metal tibial plateau, enabling flexion, extension, and rollback. Additionally, they are technically easier to implant and eliminate the risk of bearing dislocation [6]. However, fixed-bearing designs demonstrate reduced conformity during flexion and may result in concentrated stress patterns attributed to the flat geometry of the tibial articulating surface [7]. Conversely, in mobile-bearing implants, the PE inlay is designed to move along its frontal axis, allowing a degree of tibial rotation over the femur [8,9]. This mobility is theoretically associated with advantages such as an increased contact area, reduced contact stress, and decreased wear rates [10]. However, mobile-bearing implants are known to be technically more demanding, and inadequate alignment or ligament balancing can increase the risk of PE dislocation. The debate over which design is superior remains unresolved. Recent studies comparing mobile-bearing and fixed-bearing UKA have suggested that mobile-bearing designs may offer slight advantages in range of motion (ROM), while fixed-bearing designs exhibit better survivorship outcomes [10,11]. Despite these differences, most studies have shown that while clinical outcomes and overall survivorship are generally similar between the two designs, there are distinct differences in failure modes and timing [6,9,12,13,14]. Mobile-bearing UKA is primarily associated with early failures due to PE dislocation, whereas fixed-bearing UKA tends to experience later failures, typically caused by aseptic loosening.

However, long-term large cohort data remains significantly limited, and only a few direct comparative studies between fixed and mobile-bearing UKA with extended follow-up have been reported [3,7,13,15]. Although several meta-analyses comparing these two designs have been published, they predominantly include short-term follow-up data, limiting their ability to provide insights into long-term outcomes [6,12,16,17,18]. As a result, more extensive long-term research is warranted to determine which UKA design offers superior outcomes. We aimed to evaluate the mid- to long-term outcomes of medial UKA comparing mobile- versus fixed-bearing designs within a single institution over an average 10-year follow-up. We hypothesized that fixed-bearing UKA would demonstrate superior long-term survivorship compared to mobile-bearing designs. This comparative analysis is particularly relevant given the increasing volume of UKA procedures globally and the need for evidence-based guidance in implant selection for surgeons and patients.

## 2. Materials and Methods

### 2.1. Study Design

This was a retrospective analysis from a single institution. This study was approved by the institutional review board of our institution (AJOUIRB-DB-2025-228), which waived the requirement for informed consent from the patients owing to the retrospective nature of the study. The study population comprised patients with primary knee OA who underwent primary fixed-bearing or mobile-bearing medial UKA between June 2008 and July 2015, with at least five years of follow-up. Surgical indications followed established criteria: coronal deformity less than 15 degrees, flexion contracture less than 15 degrees, knee range of motion greater than 90 degrees, intact cruciate and collateral ligaments, and absence of inflammatory arthritis. There were no restrictions regarding body mass index (BMI) or age [2]. Implant type was determined by surgeon preference rather than patient-specific factors, and allocation was not randomized. We excluded patients with inflammatory arthropathy, post-traumatic OA, and ligament deficiencies. After applying these criteria, 81 consecutive patients were included during the period when both implant types were available, consisting of 45 patients with fixed-bearing UKA and 36 with mobile-bearing UKA.

### 2.2. Surgical Treatment

**Figure 1:**
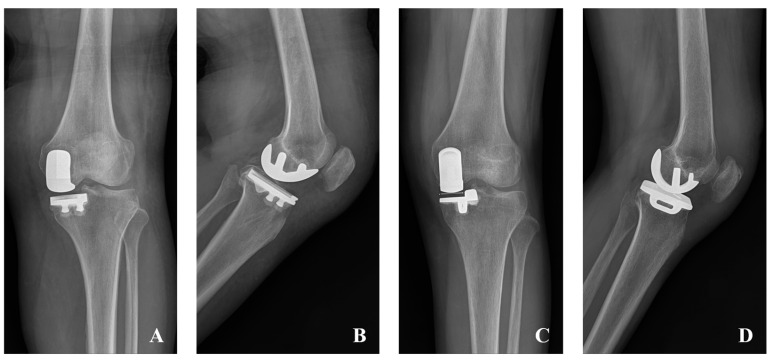
Postoperative anteroposterior (AP) (**A**) and lateral (**B**) radiographs of fixed-bearing medial unicompartmental knee arthroplasty (UKA). Postoperative AP (**C**) and lateral (**D**) radiographs of mobile-bearing medial UKA.

All procedures were performed by one senior surgeon with 15 years of experience, with UKA cases constituting more than 20% of the surgeon’s total arthroplasty volume [19]. The surgical procedure can be briefly described as follows [20]. A medial para-patella approach was used. Articular cartilage was routinely evaluated intraoperatively, and isolated medial compartment OA was confirmed. Before October 2012, the Zimmer Miller-Galante system (Zimmer Biomet, Warsaw, IN, USA) was used for fixed-bearing implants (n = 20), and the Zimmer High-Flex Knee system (Zimmer Biomet, Warsaw, IN, USA) (n = 25) was used thereafter. For mobile-bearing cases, the Oxford^®^ Partial Knee system (Zimmer Biomet, Warsaw, IN, USA) was utilized (Figure 1). Surgical technique followed the manufacturer’s instrumentation guide with conventional instruments. The proximal tibia cut was performed first, perpendicular to the mechanical axis using an extramedullary guide. Femoral cuts were subsequently made using dedicated guides. Through balancing of flexion and extension gaps, the appropriate PE insert size was selected. All components were cemented. Postoperative care included continuous passive movement ranging from 0 to 60° starting postoperative day 1 and progressing as tolerated [21]. In addition, full weight bearing with walking aids was allowed from postoperative day 1 [22].

### 2.3. Outcomes

Patient assessments and standing knee anteroposterior (AP), lateral, and Merchant radiographs, along with lower limb scanograms, were conducted before surgery, at 1, 3, and 6 months following the surgery, and yearly thereafter. Clinical outcomes were evaluated through the Western Ontario and McMaster Universities Osteoarthritis Index (WOMAC) score and knee ROM. Radiological analysis included the hip-knee-ankle axis angle (HKA) and OA progression of the contralateral compartment and patellofemoral compartment according to the Kellgren–Lawrence (K-L) grading system. K-L grading was categorized as follows: grade one—doubtful narrowing of joint space and possible osteophytic lipping; grade two—definite osteophytes and possible narrowing of joint space; grade three—moderate multiple osteophytes, definite narrowing of joint space, and some sclerosis with possible deformity of bone ends; and grade four—large osteophytes, marked narrowing of joint space, severe sclerosis, and definite deformity of bone ends [23]. All radiographic measurements were performed using the Picture Archiving and Communication Systems (PACS: Carestream Health, Rochester, NY, USA).

Failure was defined as the need for TKA conversion or PE exchange. Patients who experienced early failure and required conversion within five years were excluded from the clinical and radiologic outcome analysis. Implant survivorship was analyzed using the Kaplan–Meier survival analysis.

### 2.4. Statistical Analyses

Continuous data were analyzed using independent samples *t*-tests for comparisons between the two groups after confirming normality with the Shapiro–Wilk test. Categorical variables were examined using chi-square tests or Fisher’s exact test as appropriate. All statistical computations were conducted with R statistical software (version 3.6.3), with statistical significance defined as *p* < 0.05.

### 2.5. Intraobserver and Interobserver Reliability

Measurement reliability was evaluated through intraclass correlation coefficients (ICC) for quantitative data and linear weighted kappa coefficients for categorical measurements. Two independent evaluators (S.L and J.S) performed blinded evaluations, with the primary observer repeating measurements after a four-week interval to determine intraobserver consistency. Analysis utilized the initial measurement from the primary observer for categorical data, whereas continuous measurements represented the mean of both observers’ values. Agreement interpretation followed established criteria: ICC and kappa values ≤ 0.2 indicated poor reliability, 0.21–0.40 fair reliability, 0.41–0.60 moderate reliability, 0.61–0.80 good reliability, and >0.80 excellent reliability. Radiographic measurement reliability demonstrated intraobserver ICC and kappa coefficients ranging from 0.840 to 0.968, with interobserver coefficients spanning 0.712 to 0.928, confirming good to excellent reproducibility.

## 3. Results

A total of 81 consecutive patients were included in this study, comprising 45 patients with fixed-bearing UKA and 36 with mobile-bearing UKA. Baseline demographic characteristics, including age, follow-up duration, body mass index, sex distribution, and laterality, were comparable between the fixed-bearing and mobile-bearing groups, with no significant differences observed (Table 1). There were no statistical differences in radiological and clinical outcomes between the two groups regarding ROM, WOMAC score, contralateral OA progression, patellofemoral OA progression, or HKA (Table 2 and Table 3).

**Figure 2:**
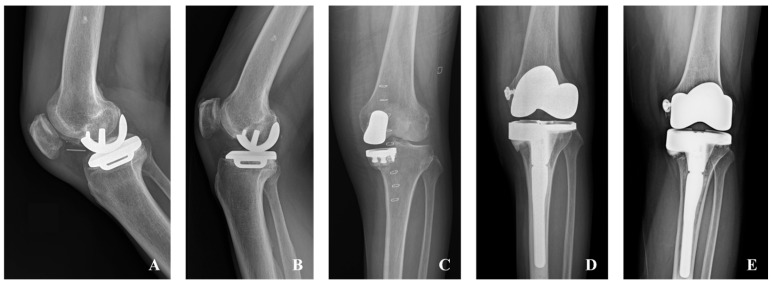
A 59-year-old male underwent mobile-bearing medial unicompartmental knee arthroplasty (UKA) on the right knee. Polyethylene (PE) dislocation occurred two years postoperatively (**A**). PE exchange was performed, and the implant has been well-maintained for nine years after the initial surgery (**B**). A 63-year-old female underwent a left knee fixed-bearing medial UKA and developed a periprosthetic tibial fracture two weeks postoperatively (**C**). Revision to total knee arthroplasty (TKA) was performed (**D**), and the TKA has been well-maintained for 11 years (**E**).

Early failure was defined as occurring within two years postoperatively, while late failure was defined as occurring after two years. In the fixed-bearing group, one patient required conversion to TKA due to a perioperative fracture (early failure). In the mobile-bearing group, six patients required TKA conversions (two early failures and four late failures) due to infection (one case), periprosthetic fracture (one case), aseptic loosening (one case), OA progression (one case), and a combination of aseptic loosening and PE dislocation (two cases). Additionally, two patients underwent PE exchanges due to PE dislocation (one early failure and one late failure) (Figure 2). Significantly higher failure rates were observed in the mobile-bearing group, both when considering conversion only (*p* = 0.041) and when including conversion or PE exchange (*p* = 0.009) (Table 4).

**Figure 3:**
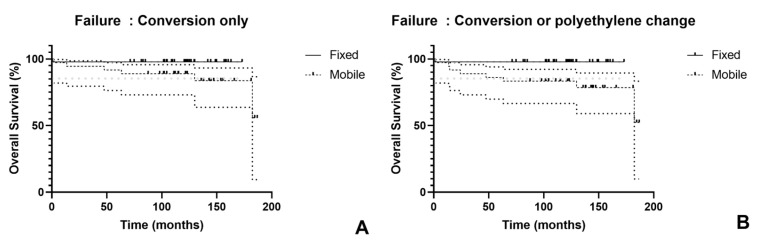
Kaplan–Meier survivorship curves comparing mobile-bearing and fixed-bearing unicompartmental knee arthroplasty. (**A**) Survival analysis with conversion to total knee arthroplasty (TKA) as the endpoint; (**B**) Survival analysis with conversion to TKA or polyethylene exchange as the endpoint. Gray dots represent 95% confidence intervals for the fixed-bearing group; black dots represent 95% confidence intervals for the mobile-bearing group.

At 10 years, survivorship was 97.8% for fixed-bearing compared to 88.9% for mobile-bearing implants using conversion as the sole failure criterion, which did not reach statistical significance (*p* = 0.066). When failure included both conversion and PE exchange, 10-year survivorship remained 97.8% for fixed-bearing while declining to 83.3% for mobile-bearing designs, representing a significant difference (*p* = 0.015) (Figure 3).

## 4. Discussion

Key findings from this study demonstrated comparable WOMAC scores, ROM, and radiological outcomes between mobile and fixed-bearing UKA. However, fixed-bearing implants exhibited significantly lower failure rates and better survivorship than mobile-bearing UKA. The 10-year survivorship was 97.8% for fixed-bearing UKA compared to 88.9% (failure defined as conversion only) or 83.3% (failure defined as conversion or PE exchange) for mobile-bearing UKA. These results showing no differences between bearing types, except for survivorship, were consistent with most previous studies [6,9,12,13,14].

Ten-year survivorship after medial UKA varies across studies, ranging from 89 to 98% for fixed-bearing designs and from 82 to 95% for mobile-bearing designs across case series, comparative studies, registry data, and meta-analyses, making direct comparison challenging due to this heterogeneity [3,10,11,13,15,24,25,26]. However, direct comparative studies reporting long-term survivorship are quite limited (Table 5). Emerson et al. [7] compared 51 fixed-bearing and 50 mobile-bearing UKAs and reported an 11-year survivorship of 92% for both groups when failure was defined as revision for any reason. Parratte et al. [15] conducted a study with a minimum follow-up of 15 years and reported that the 20-year survivorship was 83% for fixed-bearing UKA and 80% for mobile-bearing UKA, showing no significant long-term difference. Similarly, Neufeld et al. [13], in a study with a minimum 10-year follow-up, reported a 10-year survivorship of 90.9% for fixed-bearing UKA and 82.9% for mobile-bearing UKA, showing no significant difference. However, Tay et al. [3], through a cohort analysis of 2015 patients with an average follow-up of 8 years, found that the 15-year survivorship of cemented fixed-bearing UKA was 92%, compared to 80% for cemented mobile-bearing UKA, indicating superior long-term survivorship for the fixed-bearing design. In these direct comparative studies, fixed-bearing designs show a trend toward superior survivorship compared to mobile-ring designs.

While multiple meta-analyses have reported no significant difference in survivorship between the two designs, the studies included in these meta-analyses predominantly consist of short-term and mid-term data, with long-term studies being limited [6,12,16,17,18]. Cao et al. [12] reported that although there was no difference in overall revision rates, mobile-bearing designs tended to fail early, whereas fixed-bearing designs tended to experience late failures. Studies utilizing registry data have shown similar trends. Kannan et al. reported that the 15-year survivorship of fixed-bearing UKA was superior to that of mobile-bearing UKA based on an analysis of 50,380 medial UKAs from the Australian registry [10]. Overall, fixed-bearing UKA appears to have a slight advantage in long-term survivorship.

In our cohort, only one patient in the fixed-bearing group experienced a perioperative periprosthetic fracture, requiring revision to TKA. However, no cases of aseptic loosening were observed. Contrary to these concerns, our findings demonstrated favorable outcomes, with a 10-year survivorship of 97.8% in the fixed-bearing group. Conversely, in the mobile-bearing group, failure cases were more frequent. This group included one case of perioperative periprosthetic fracture, two cases of PE dislocation requiring PE exchange, one case of infection, two cases of aseptic loosening, and one case of OA progression leading to TKA conversion. Consequently, the 10-year survivorship for mobile-bearing UKA was 88.9% (when defining failure as conversion to TKA) or 83.3% (when including PE exchange as failure).

One possible explanation for the lower survivorship of MB implants may be related to their inherent design characteristics. While MB implants were developed to better replicate physiological knee kinematics through minimization of contact stress and constraint, which theoretically should result in reduced PE wear and implant loosening, they come with their own set of unique challenges. MB implants are sensitive to soft tissue balancing, which may predispose to bearing dislocation or impingement [9]. Increased medial compartment stress can contribute to bearing dislocation, and to prevent this, surgeons might attempt overcorrection to valgus alignment, which in turn can lead to OA progression in the lateral compartment. According to various studies, it is recommended to target mild varus alignment during medial UKA, as this has been reported to provide the best clinical outcomes and survival rates [27,28,29,30]. In our study, both groups demonstrated similar postoperative varus alignment, averaging 4.9° and 5.9°, respectively, with no significant difference between the groups. Despite the postoperative mild varus alignment, no statistical differences in OA progression were observed between the two groups. However, failure rates were higher in the mobile bearing group, suggesting that MB implants may have a narrower tolerance range for postoperative alignment, making them more sensitive to alignment variations even within the acceptable range.

Another factor is that Asian patients are reported to have higher rates of PE dislocation compared to Western patients due to regular hyperflexion and anatomical features [31]. Additionally, Sun et al. reported that among Asians, Koreans have even higher rates of PE dislocation [32]. In this study, we observed two PE dislocations that needed PE exchange, and two out of six revision cases were directly related to PE dislocation, constituting a significant proportion. Furthermore, Kang et al. reported that after changing from the Oxford design to a newer anatomical design in mobile-bearing UKA, bearing dislocation rates were significantly reduced [33]. Our study, being a Korean population-based study, suggests that these anatomical factors and lifestyle habits may significantly contribute to the lower survival rates of MB in this population.

Regarding clinical and functional outcomes after UKA, most studies report no difference between mobile-bearing and fixed-bearing UKA [6,9,12,13,14]. According to a study by Hariri et al., the mobile-bearing group showed better ROM results than the fixed-bearing group, although there was no difference in functional scores [11]. In contrast, Huang et al., through a systematic analysis, reported that fixed-bearing UKA demonstrated higher knee scores and superior ROM [18]. Theoretically, mobile-bearing implants, due to their design characteristics that more closely replicate native knee biomechanics, would be expected to achieve better ROM. However, clinical study results have provided insufficient evidence to support this hypothesis. Consistent with most studies, our study also found no significant differences in clinical outcomes, including ROM, between the two groups.

Future research should focus on several key areas to further elucidate the optimal bearing design for UKA. First, well-designed randomized controlled trials with larger sample sizes and extended follow-up periods exceeding 15 years are needed to definitively establish the long-term superiority of one design over the other. Second, investigation into the biomechanical factors contributing to bearing dislocation in Asian populations, particularly focusing on knee kinematics during deep flexion activities, could help develop design modifications or surgical techniques to reduce this complication. Third, studies evaluating newer generations of mobile-bearing implants with improved design features, such as enhanced constraint mechanisms or anatomical geometries, may demonstrate better outcomes compared to earlier designs used in our study. Additionally, research examining patient-specific factors that predict success with each bearing type could enable more personalized implant selection, taking into account age, activity level, anatomical variations, and cultural lifestyle factors, potentially improving overall outcomes and survivorship rates across diverse patient populations.

Several limitations should be acknowledged in this study that may affect the interpretation and generalizability of our findings. First, the study employed a retrospective design without randomization of the two groups, and implant selection was performed without established criteria, potentially introducing selection bias. Second, the use of different fixed-bearing implant types and the associated learning curve with new implant systems may have introduced confounding variables that could have influenced the outcomes. However, since all UKAs were performed by one single senior surgeon with consistent surgical techniques, we believe these differences would have less impact on the overall results. Third, since this study is based on a Korean population, the results may be associated with the anatomical characteristics and lifestyle habits of Asian patients, which may limit the generalizability of our findings to Western populations.

Despite these limitations, the present study has several notable strengths that enhance the validity and reliability of our findings. First, all surgical procedures were performed by a single experienced surgeon with more than 15 years of expertise in unicompartmental knee arthroplasty, ensuring consistency in surgical technique and implant positioning across both groups. This uniformity minimizes the potential confounding effects of varying surgical skills and technical approaches that often occur in multi-surgeon studies. Second, our study provides valuable long-term data with a mean follow-up period of 10.6 years, which is particularly important given that many bearing-related complications and late failures may not become apparent in short-term or mid-term studies. Third, the consecutive patient enrollment and consistent application of surgical indications throughout the study period reduce selection bias and enhance the generalizability of our results within similar patient populations, particularly in Asian populations with comparable anatomical characteristics and lifestyle patterns.

## 5. Conclusions

At a mean 10.6-year follow-up, clinical and radiological outcomes were comparable, but fixed-bearing UKA demonstrated superior survivorship.

## Figures and Tables

**Table 1:** The demographic data.

	Total (n = 81)	Fixed (n = 45)	Mobile (n = 36)	*p*-Value
Age (years)	57.2 ± 6.4	57.5 ± 6.6	56.8 ± 6.2	0.596
Follow-up period (months)	127.0 ± 28.6	121.6 ± 27.0	133.9 ± 29.5	0.056
Height (cm)	158.2 ± 8.3	158.0 ± 8.3	158.5 ± 8.5	0.770
Weight (kg)	66.1 ± 10.4	65.8 ± 9.7	66.5 ± 11.3	0.753
BMI (kg/m^2^)	26.3 ± 3.1	26.3 ± 3.1	26.4 ± 3.1	0.945
Sex (M/F)	17/64	7/38	10/26	0.286
Side (L/R)	46/35	28/17	18/18	0.380

BMI, body mass index; M, male; F, female; L, left; R, right.

**Table 2:** Clinical outcomes in the two groups.

	Total (n = 77)	Fixed (n = 44)	Mobile (n = 33)	*p*-Value	Mean Difference [95% CI]
ROM (°) (preoperative)	145.5 ± 18.3	144.4 ± 22.2	146.8 ± 11.2	0.540	2.4 [−6.0, 10.8]
ROM (°) (postoperative)	144.6 ± 13.6	143.4 ± 14.7	146.1 ± 12.0	0.386	2.7 [−3.4, 8.7]
ROM (°) (delta)	−0.9 ± 23.4	−1.0 ± 27.6	−0.8 ± 16.6	0.958	0.2 [−9.8, 10.4]
WOMAC score (postoperative)	28.2 ± 20.8	27.6 ± 21.8	28.9 ± 19.7	0.791	1.3 [−8.3, 10.8]

CI, confidence interval; ROM, range of motion; WOMAC, Western Ontario and McMaster Universities Osteoarthritis Index.

**Table 3:** Radiologic outcomes in the two groups.

	Total (n = 77)	Fixed (n = 44)	Mobile (n = 33)	*p*-Value
Contralateral K-L grade (Pre.) grade (0/1/2)	8/52/17	5/31/8	3/21/9	0.628
Contralateral K-L grade (Post.) grade (1/2/3/4)	25/47/4/1	14/27/3/0	11/20/1/1	0.600
Contralateral K-L grade (delta) grade (0/1/2)	32/41/4	16/26/2	16/15/2	0.495
PFJ K-L grade (Pre.) grade (0/1/2)	7/46/24	5/29/10	2/17/14	0.167
PFJ K-L grade (Post.) grade (1/2/3/4)	1/34/38/4	0/23/18/3	1/11/20/1	0.182
PFJ K-L grade (delta) grade (0/1/2)	50/26/1	26/17/1	24/9/0	0.366
HKA (°) (Pre.)	173.1 ± 4.3	173.0 ± 4.6	173.2 ± 4.0	0.795
HKA (°) (Post.)	174.6 ± 4.9	174.1 ± 5.1	175.1 ± 4.6	0.378
HKA (°) (delta)	1.5 ± 3.8	1.2 ± 3.9	1.9 ± 3.9	0.417

K-L, Kellgren–Lawrence; PFJ, patellar–femoral joint; HKA, hip-knee-ankle angle; Pre, preoperative; Post, postoperative.

**Table 4:** Failures in the two groups.

	Total (n = 81)	Fixed (n = 45)	Mobile (n = 36)	*p*-Value
Failure (TKA conversion)	7 (8.6%)	1 (2.2%)	6 (16.7%)	**0.041**
Early/Late	3/4	1/0	2/4	
Failure (TKA conversion or PE exchange)	9 (11.1%)	1 (2.2%)	8 (22.2%)	**0.009**
Early/Late	4/5	1/0	3/5	

Bold values indicate statistically significant values (*p* < 0.05). TKA, total knee arthroplasty; PE, polyethylene.

**Table 5:** Long-term survivorship comparison of mobile- versus fixed-bearing UKA: Summary of direct comparative studies.

Authors	Year	Bearing	No. of Knees	Mean Follow-Up (Years)	Survivorship
Emerson et al. [7]	2002	FB	51	7.7	11 years: 92%
		MB	50	6.8	11 years: 92%
Parratte et al. [15]	2012	FB	79	17.2	20 years: 83%
		MB	77	17.2	20 years: 80%
Neufeld et al. [13]	2018	FB	68	11.5	10 years: 90.9%
		MB	38	14.2	10 years: 82.9%
Tay et al. [3]	2023	FB	450	7.0	15 years: 92%
		MB	496	11.3	15 years: 80%
Lim et al. (current study)	2025	FB	45	10.1	10 years: 97.8%
		MB	36	11.2	10 years: 88.9% (conversion to TKA) 10 years: 83.3% (conversion to TKA + PE exchange)

FB, fixed-bearing; MB, mobile-bearing; TKA, total knee arthroplasty; PE, polyethylene.

## Data Availability

Data are unavailable due to privacy or ethical restrictions.
